# Modelling gestational weight gain trajectories and risk of adverse birth outcomes using super imposition by translation and rotation: findings from two Brazilian cohort studies

**DOI:** 10.1016/j.lana.2026.101561

**Published:** 2026-07-09

**Authors:** Audêncio Victor, Liania A. Luzia, Tamiris Ramos Silva, Aldaisa Pereira Lopes, Rebecca E. Penzias, Isabel Oliveira Aires, Patrícia Helen Rondó, Eric O. Ohuma

**Affiliations:** a Public Health Postgraduate Program, School of Public Health, University of São Paulo, Av. Dr. Arnaldo, 715 - Cerqueira César, São Paulo, SP, 01246-904, Brazil; b Nutrition Department, School of Public Health, University of São Paulo, Av. Dr. Arnaldo, 715 - Cerqueira César, São Paulo, SP, 01246-904, Brazil; c Faculty of Epidemiology and Population Health, London School of Hygiene & Tropical Medicine, Keppel Street, London, WC1E 7HT, UK

**Keywords:** Gestational weight gain, SITAR model, Growth trajectories, Maternal health, Brazil, Epidemiology

## Abstract

**Background:**

Gestational weight gain (GWG) is a key determinant of maternal and neonatal health, yet there are no clear recommendations for tracking GWG trajectories, beyond total amount, in routine care. We aimed to characterise GWG trajectories in terms of both amount and velocity and examine their associations with adverse neonatal outcomes in two Brazilian cohorts, using INTERGROWTH-21st (IG-21st) centiles.

**Methods:**

We analysed 3770 pregnant women in two population-based cohorts in São Paulo state, Brazil: Jundiaí (n = 965; 1997–2000; median age 24 years [IQR 20–29]; 21% non-White) and Araraquara (n = 2805; 2017–2024; median age 27 years [IQR 22–32]; 54% non-White). GWG trajectories were modelled using the Super Imposition by Translation and Rotation (SITAR) method. Predicted GWG amount and velocity were classified against IG-21st 25th and 75th centiles for women with normal weight. Associations with low birthweight (LBW), macrosomia, preterm birth (PTB), and 5-min Apgar score <7 were estimated using multivariable Poisson regression with robust variance estimation.

**Findings:**

SITAR explained 85.6% of GWG variance in Jundiaí and 84.0% in Araraquara. GWG below the 25th centile was associated with LBW (RR 2.14; 95% CI 1.47–3.11) and PTB (RR 1.95; 95% CI 1.20–3.17); above the 75th centile, with macrosomia (RR 2.58; 95% CI 1.40–4.77). GWG velocity above the 75th centile was strongly associated with macrosomia (RR 3.31; 1.58–6.92).

**Interpretation:**

SITAR provided a parsimonious description of GWG trajectories and identified clinically relevant deviations in amount and velocity. IG-21st 25th and 75th centiles offer risk stratification identifying those most at risk of poor neonatal outcomes.

**Funding:**

São Paulo Research Foundation (FAPESP).

**Research in context**

**Evidence before this study**

We searched PubMed/MEDLINE on 25 February 2026, from inception with no start- date restriction, using the strategy: (“gestational weight gain” OR “GWG” OR “pregnancy weight gain”) AND (“trajectory” OR “longitudinal modelling” OR “growth curve” OR “SITAR”) AND (“neonatal outcomes” OR “low birthweight” OR “preterm birth” OR “macrosomia”), with “Brazil” as an optional term. The same structure was adapted to Embase, Web of Science, and LILACS, combining controlled vocabulary (MeSH, Emtree) with title/abstract free-text terms. We identified only one published study applying SITAR to model GWG trajectories (Riddell et al., 2017), in a North American cohort, which did not evaluate how SITAR-derived parameters translate into clinically meaningful categories. To our knowledge, no previous study has applied SITAR to GWG trajectories in a Latin American population. Both inadequate and excessive GWG are associated with adverse outcomes such as low birthweight (LBW), preterm birth (PTB), macrosomia, and postpartum complications. Current recommendations, particularly from the Institute of Medicine (IOM), rely mainly on total weight-gain thresholds by pre-pregnancy body mass index (BMI) and do not account for the longitudinal, dynamic nature of gain across gestation. The INTERGROWTH-21st (IG-21st) project developed gestational-age-specific GWG centiles from a prospective multi-ethnic cohort including Brazil, providing an international reference for women with normal pre-pregnancy BMI, but their integration with longitudinal modelling remains limited. The SITAR (Super-Imposition by Translation and Rotation) model yields interpretable parameters of size and velocity and is widely used in paediatric growth research; its application to GWG is recent and scarce.

**Added value of this study**

We applied SITAR to longitudinal GWG data from two population-based Brazilian cohorts conducted about two decades apart, providing one of the first trajectory-based descriptions of GWG dynamics in Latin America using a reproducible growth-curve framework. Parsimonious models using size and velocity captured most of the variation in GWG in both cohorts. We observed cohort differences in the timing of peak GWG velocity, suggesting temporal shifts in pregnancy weight gain dynamics in Brazil. Mapping SITAR-predicted size and velocity to IG-21st centiles, deviations from the central range were consistently related to neonatal outcomes: lower-than-expected GWG was linked to higher risk of LBW and PTB, whereas higher-than-expected and especially faster gain was linked to macrosomia, indicating that velocity carries information beyond total gain.

**Implications of all the available evidence**

Taken together, available evidence supports moving beyond total GWG alone and considering the rate of gain during pregnancy as a complementary marker of risk. In routine antenatal care, monitoring GWG velocity may provide earlier warning signals for both fetal growth restriction-related outcomes and macrosomia. For women with normal pre-pregnancy BMI, the IG-21st, 25th, and 75th centiles provide pragmatic thresholds for interpreting trajectory-derived measures in a standardised way. Future studies should assess generalisability across BMI categories, evaluate early-pregnancy prediction, test whether incorporating velocity improves clinical algorithms, and develop reference standards for GWG velocity.

## Introduction

Gestational weight gain (GWG) is a modifiable factor with direct implications for maternal, fetal and neonatal health.^1^, ^2^, ^3^, ^4^, ^5^, ^6^, ^7^, ^8^, ^9^, ^10^ With the growing increase in obesity worldwide, particularly among women of reproductive age,^11^, ^12^, ^13^, ^14^ managing GWG has become increasingly important. Inadequate or excessive GWG increases the risk of maternal complications such as hypertension, gestational diabetes, caesarean delivery, and postpartum weight retention, while also negatively affecting the fetus, leading to intrauterine growth restriction, low birthweight (LBW), preterm birth (PTB), perinatal mortality, macrosomia, childhood obesity, and cardiometabolic issues.^1^^,^^5^, ^6^, ^7^, ^8^, ^9^, ^10^^,^^15^, ^16^, ^17^, ^18^, ^19^ Effective GWG management can reduce these complications and improve perinatal and infant outcomes.^20^ Several factors contribute to excessive or insufficient GWG, including socioeconomic factors such as income and education,^19^^,^^21^ behavioural factors like physical activity, smoking, and alcohol consumption,^22^^,^^23^ and maternal biological factors like pre-gestational (Body Mass Index) BMI, age, and parity.^24^, ^25^, ^26^, ^27^, ^28^ Psychological and health factors, such as social support, sleep deprivation, and conditions like diabetes and hypertension, also play an important role.^2^^,^^19^^,^^29^

Global studies show significant variations in GWG by region, with estimates ranging from 5.4 kg (kg) in Sub-Saharan Africa to 13.0 kg in Central and Eastern Europe.^30^ On average, high-income countries GWG is 9.8 kg compared to 5.3 kg in low-income countries.^30^ In Brazil, GWG has increased over the years, particularly among low-income women.^31^ Data from the Pelotas birth cohorts (1982–2015) show that although mean GWG remained relatively stable over time (11.8 kg in 1982 vs. 12.0 kg in 2015), the proportion of women with excessive GWG increased from 24.6% in 1982 to 35.7% in 2015, based on the recommendations of the Institute of Medicine (IOM).^32^ Similarly, more recent studies, such as the LINDA-Brazil (2014–2017),^33^ and Araraquara cohorts, report a high prevalence of excessive GWG according to IOM, reflecting an ongoing challenge in weight management during pregnancy.^34^^,^^35^ These data highlight the growing challenge of controlling weight during pregnancy.

In response to these challenges, international efforts have been made to standardise the classification of GWG. Since the 1990s, the IOM recommendations have been used to classify GWG, but they have limitations, as they were based on cross-sectional studies conducted exclusively in the United States.^36^, ^37^, ^38^ In 2016, the Intergrowth-21st (IG) standards were developed with data from a multi-ethnic cohort, including Brazil, but they are limited to women with normal BMI and only begin monitoring from the 14th week of gestation, limiting their applicability in the first trimester.^39^ This also raises the broader question of whether GWG standards should be strictly prescriptive based on healthy reference populations or adapted to existing BMI distributions. In Brazil, the Ministry of Health adopted a national GWG curve in 2022, based on data from diverse regions of the country. However, a key limitation of these curves is that they were derived from secondary analyses of existing datasets, rather than from prospective studies specifically designed to develop GWG standards.^40^

The Super Imposition by Translation and Rotation (SITAR) model offers a unique opportunity to complement existing guidelines by providing a more detailed and personalised analysis by modelling not just the amount of weight gain, but also the rate of weight gain.^41^^,^^42^ SITAR has already been demonstrated and utilised for describing infant growth curves.^43^, ^44^, ^45^ This study applied SITAR modelling to (i) characterise GWG trajectories in terms of both the amount and velocity in the overall sample, and (ii) examine associations between these trajectory features and neonatal outcomes in two Brazilian cohorts. In addition, amount and velocity of weight gain were interpreted using the IG-21st GWG references for women with normal pre-pregnancy BMI, with the 25th and 75th centiles selected as thresholds to define lower and higher-than-expected weight gain that might be associated with adverse neonatal outcomes.

## Methods

### Study design

This is an observational epidemiological cohort study comparing data from two cohorts (Araraquara and Jundiaí) separated by a 20-year interval and conducted in municipalities in São Paulo state, Brazil. The Araraquara cohort included women with a gestational age ≤19 weeks who received prenatal care at 37 Health Units and the Special Health Service (SESA) in Araraquara, São Paulo, Brazil. The participants were followed quarterly during pregnancy until the birth of their children, from 2017 to 2024, at the “Gota de Leite” Municipal Maternity. All participants were beneficiaries of the Unified Health System (SUS), which predominantly serves low-income families. Women with multiple pregnancies, abortions, or missing information on height, pre-pregnancy weight, and birth weight were excluded.^35^

The Jundiaí cohort (USP-MatStress) initially included 1182 women who received prenatal care between September 1997 and August 2000 in 12 health units and five hospitals in Jundiaí, São Paulo, Brazil. Of these, 965 women were followed in a cohort study before the 16th week of pregnancy until the birth of their children.^46^

GWG was calculated as the difference between weight measured at each prenatal visit and self-reported pre-pregnancy weight. Repeated weight measurements were recorded across pregnancy, typically once per trimester, enabling longitudinal assessment of maternal weight trajectories. Gestational age was estimated by ultrasound. Although women contributed multiple weight measurements, GWG classification was not performed at each visit. Instead, individual GWG trajectories were modelled using the SITAR approach, and predicted GWG size and velocity were extracted at a single pre-specified reference gestational week (36 weeks) for each woman, providing one exposure measure per pregnancy. Week 36 was chosen because it corresponds to the last gestational-age window of routine weight measurement in both cohorts (cohort data truncated at 36 weeks); beyond this point, extraction would rely on extrapolation. Although the INTERGROWTH-21st GWG standard extends to week 40, mean weekly weight gain decelerates after 34 weeks (0.37 vs. 0.52–0.57 kg/week earlier), so that approximately 92% of the total GWG has already been achieved by week 36. Predicted values were classified relative to the IG centiles, with the 25th and 75th centiles selected as thresholds because they align with the IOM recommendations for women with normal pre-pregnancy weight the weight gain between the 25th and 75th IG centiles at term (10.9–17.9 kg) was comparable with the IOM recommendation (11.5–16.0 kg) and with the optimal GWG reported in a secondary analysis of the INTERBIO-21st Fetal Study.^10^ Because the INTERGROWTH-21st GWG standard applies only to women with normal pre-pregnancy BMI, the adjusted Poisson analyses comparing P25–P75 with <P25 and >P75 categories were restricted to participants with pre-pregnancy BMI 18.5–<25 kg/m^2^.^47^ The initial eligibility criterion was ≤12 weeks of gestation but was extended to 19 weeks due to delayed initiation of prenatal care among a substantial proportion of women.

### Maternal characteristics

Various factors were considered as covariates in the regression analyses, including socioeconomic and demographic characteristics: age (years), education (years), number of people per room (defined as the number of people living in the household per room), gravidity (total number of pregnancies), parity, race/ethnicity, and marital status (married/in a stable union, single/separated/widowed). Race/ethnicity was self-reported by participants at enrolment according to the five categories used by the Brazilian Institute of Geography and Statistics (IBGE): White, Black, Brown/mixed, Asian, and Indigenous; for analysis, these were categorised into a binary variable (White vs. non-White), with non-White comprising the Black, Brown/mixed, Asian, and Indigenous categories. Lifestyle factors such as smoking and alcohol consumption were also considered, as well as morbidities (diabetes, hypertension, urinary tract infection). Anthropometric measures included body mass index (BMI), gestational age, and weight. These covariates were included in the adjusted robust Poisson regression models used to estimate risk ratios, whereas the SITAR models were used only to derive GWG trajectories.

### Neonatal outcomes

LBW was defined as a birth weight of less than 2500 g, and PTB as birth before 37 completed weeks of gestation. Macrosomia was defined as a birth weight of 4000 g or more. The Apgar score at 5 min was categorised as a binary variable: <7 and ≥7. Apgar scores <7 were considered indicative of potential neonatal distress, reflecting compromised cardiopulmonary and neurological function that requires clinical attention. These outcomes were pre-specified during cohort harmonisation based on three criteria: routine recording with identical definitions in both cohorts, consistent use in the prior GWG literature,^1^, ^2^, ^3^ and representation of distinct physiological pathways of fetal growth (LBW, macrosomia), timing of delivery (PTB), and immediate neonatal adaptation (Apgar at 5 min).

### Ethical issues

The Araraquara study was approved by the Research Ethics Committee on Human Subjects at the School of Public Health, University of São Paulo, prior to data collection (protocol CAEE: 59787216.2.0000.5421). The Jundiaí study was approved by the same committee together with the Health Secretariat of Jundiaí, SP (protocol 289/98). In both cohorts, written informed consent was obtained from all participants before enrolment.

### Statistical analysis

Descriptive statistics were used for data analysis. Median and interquartile range (IQR) were presented for continuous variables, and frequencies (n) and percentages (%) for categorical variables. To model the GWG trajectories, the SITAR model was employed. The SITAR model estimates a population–average curve for GWG and adjusts for individual variation in GWG, including the amount, timing, and rate of weight gain. These parameters can be used to investigate associations with adverse outcomes, such as the impact of early onset weight gain on maternal and infant health.

SITAR is a nonlinear mixed-effects approach and is expressed using the following equation:

$${\;y}_{it}=\alpha_{i}+h\left(\frac{t\;-\;\beta_{i}}{\exp\left(\;-\;c_{i}\right)}\right)\;+\;g_{it}$$

The SITAR model estimates individual weight gain trajectories by aligning each curve to the average curve through three key parameters ($\alpha_{i}$: vertical adjustment amount; $\beta_{i}\text{:}$ horizontal adjustment timing and $c_{i}\text{:}$ slope adjustment rate of weight gain). In this approach, $y_{it}$ represents the weight gain measure for woman i at gestational age t, and $g_{it}$ are the error terms, which are assumed to be normally distributed. The function $h_{t}$ is a nonlinear function of gestational age, typically modelled by a spline that describes the relationship between weight gain and gestational age, representing the best–fit curve. The objective is to determine the parameters' values ci, such that the individual curves align as closely as possible with the average curve $h_{t}$. These parameters encompass fixed and random effects, allowing each woman's curve to deviate from the population average in three ways. For example, the parameter $\alpha_{i}$ can be expressed as $\alpha_{i}=\alpha_{0}+\alpha_{1}$, where $\alpha_{0}$ is estimated average weight gain at the conception and $\alpha_{1i}$ represent the individual derivation, acting as a vertical shift on the average curve and corresponding to the absolute weight gain. Although $\alpha_{i}$ it should theoretically equal zero (as weight gain starts at 0 kg), allowing variation in $\alpha_{i}$ capture measurement errors from self-reported pre-gestational weight and improving model fit, thus reflecting a combination of measurement error and possible model inadequacies. Additionally, the parameter $\beta_{i}$ allows horizontal shifting of the average curve, adjusting for the timing of weight gain, while the parameter $c_{i}$ modifies the curve’s slope, capturing the acceleration of weight gain.

The model selection was based on the Akaike Information Criterion (AIC), complemented by the Bayesian Information Criterion (BIC) and the percentage of variance explained. Models with lower AIC and BIC values and a higher percentage of variance explained were considered superior.^48^ All analyses were performed using the sitar package (version 1.0.3)^49^ in R software (version 4.1.0, R Foundation for Statistical Computing, Vienna, Austria).

To enable meaningful interpretation and comparability with international standards, individual GWG trajectories were first modelled using the SITAR approach, which generated smooth predicted values of amount and velocity weight gain across gestation for each woman. From these fitted trajectories, predicted GWG (amount and velocity) were extracted at a standard reference gestational week for each participant. These model-derived estimates were then classified according to IG centiles for women with normal pre-pregnancy BMI.^47^ Using IG-based thresholds, participants were categorised as below the 25th centile (<P25), between the 25th and 75th centiles (P25–P75), or above the 75th centile (>P75) for both amount (kg) and velocity (kg/week), with P25–P75 used as the counterfactual.

Associations between IG-classified GWG parameters and neonatal outcomes were estimated using Poisson regression with a log link and robust variance to obtain risk ratios (RRs) and 95% confidence intervals.^50^ Models were adjusted for maternal age, pre-pregnancy BMI, education, race, parity, gestational diabetes, hypertensive disorders, smoking, alcohol, and infant sex. Smoking and alcohol referred to current use in the first trimester (self-reported at the first prenatal visit); gestational diabetes and hypertensive disorders were coded as first-trimester indicators. SITAR models were fitted with random effects for size (a) and velocity (c); the timing (b) random effect was not included as the three-parameter specification failed to converge in our data. Outliers were inspected via residual diagnostic plots (Supplementary Fig. S3); implausible values flagged during data cleaning were verified against original records before modelling. Regression analyses used complete cases; SITAR used all available weight measurements per participant within a mixed-effects framework.

### Role of the funding source

The funders of the study had no role in study design, data collection, data analysis, data interpretation, writing of the report, or the decision to submit the paper for publication. The corresponding author had full access to all data in the study and final responsibility for the decision to submit it for publication.

## Results

A total of 3770 pregnant women were included, comprising 2805 from the Araraquara cohort and 965 from the Jundiaí cohort, located in São Paulo, Brazil (Table 1, Supplementary Figs. S1 and S2). The sociodemographic and clinical characteristics of the participants are presented in Table 1. Women in the Araraquara cohort had a higher median age (27 years; IQR: 22–32) compared to those in Jundiaí (24 years; IQR: 20–29), as well as a higher pre-pregnancy BMI (25.8 vs. 22.5 kg/m^2^) and greater body weight across all trimesters of pregnancy. A higher proportion of non-White women was also observed in Araraquara (54%) compared to Jundiaí (21%). Conversely, the proportion of adolescents was higher in Jundiaí (24%) than in Araraquara (9.1%). Higher education (>12 years of schooling) was more prevalent in Araraquara (8.7%) than in Jundiaí (2.9%). Additionally, Araraquara had a higher household density (33% in the upper tertile of persons per room), a greater prevalence of primigravida women (39%), and a higher average mid-upper arm circumference (29.7 cm vs. 27.0 cm). Adverse maternal conditions were also more frequent in Araraquara, particularly gestational hypertension and diabetes. The prevalence of diabetes in the third trimester was 11% in Araraquara, compared to just 0.2% in Jundiaí. Urinary tract infections in the first trimester were reported in 11% of women in Araraquara and 6.1% in Jundiaí (Table 1).

**Table 1:** Characteristics of pregnant women in Araraquara and Jundiai Cohort Studies, São Paulo, Brazil.

Variables	N	Cohorts		
		Overall N = 3770	Araraquara N = 2805	Jundiai N = 965
Age (Year)^a^	3733	26 (22–31)	27 (22–32)	24 (20–29)
Missing		37	37	0
Age (Group)^b^	3733			
≤19		484 (13%)	251 (9.1%)	233 (24%)
20–35		2747 (74%)	2092 (76%)	655 (68%)
≥35		502 (13%)	425 (15%)	77 (8.0%)
Missing		37	37	0
Education (years)	3701			
>12		268 (7.2%)	241 (8.7%)	27 (2.9%)
≤12		3433 (93%)	2523 (91%)	910 (97%)
Missing		69	41	28
Number of people per room (tertile)	3729			
1st		1374 (37%)	1021 (37%)	353 (37%)
2nd		1286 (34%)	844 (31%)	442 (46%)
3rd		1069 (29%)	899 (33%)	170 (18%)
Missing		41	41	0
Race	3730			
Non-white		1709 (46%)	1503 (54%)	206 (21%)
White		2021 (54%)	1262 (46%)	759 (79%)
Missing		40	40	0
Marital status	3730			
Married or in a stable relationship		3150 (84%)	2427 (88%)	723 (75%)
Single, separated, or widowed		580 (16%)	338 (12%)	242 (25%)
Missing		40	40	0
Smoking	3620	345 (9.5%)	222 (8.1%)	123 (14%)
Missing		150	50	100
Alcohol	3620	734 (20%)	545 (20%)	189 (22%)
Missing		150	50	100
Parity	3712			
1 previous pregnancy		1194 (32%)	780 (28%)	414 (43%)
2 or more previous pregnancies		1453 (39%)	902 (33%)	551 (57%)
Primigravida		1065 (29%)	1065 (39%)	0 (0%)
Missing		58	58	0
Arm circumference (cm)	3464	28.7 (26.0–32.5)	29.7 (26.6–33.4)	27.0 (25.0–29.4)
Missing		306	306	0
Hemoglobin (mg/dL)	3183	12.50 (11.90–13.20)	12.50 (11.90–13.10)	12.60 (11.90–13.40)
Missing		587	587	0
Diabetes				
1st Trimester	3685	139 (3.8%)	136 (4.9%)	3 (0.3%)
Missing		85	52	33
2nd Trimester	2870	147 (5.1%)	144 (7.3%)	3 (0.3%)
Missing		900	828	72
3rd Trimester	2631	184 (7.0%)	182 (11%)	2 (0.2%)
Missing		1139	1102	37
Hypertension				
1st Trimester	3681	228 (6.2%)	193 (7.0%)	35 (3.8%)
Missing		89	53	36
2nd Trimester	2871	186 (6.5%)	140 (7.1%)	46 (5.1%)
Missing		899	828	71
3rd Trimester	2634	214 (8.1%)	134 (7.9%)	80 (8.6%)
Missing		1136	1102	34
Urinary tract infection				
1st Trimester	3669	359 (9.8%)	303 (11%)	56 (6.1%)
Missing		101	52	49
2nd Trimester	2854	202 (7.1%)	138 (7.0%)	64 (7.3%)
Missing		916	828	88
3rd Trimester	2626	181 (6.9%)	102 (6.0%)	79 (8.6%)
Missing		1144	1102	42
Gestational age				
1st Trimester	3442	13.3 (11.3–15.3)	13.9 (12.4–15.7)	11.0 (8.3–13.0)
Missing		328	296	32
2nd Trimester	2838	24.29 (22.71–25.86)	23.86 (22.43–25.29)	25.43 (23.71–27.14)
Missing		932	856	76
3rd Trimester	2668	33.43 (32.14–34.86)	33.14 (31.86–34.43)	34.14 (32.43–35.71)
Missing		1102	1059	43
Pre-gestational BMI (kg/m^2^)^a^	3407	24.8 (21.5–29.2)	25.8 (22.3–30.7)	22.5 (20.7–25.1)
Missing		363	327	36
Weight (kg)				
1st Trimester	3473	65 (56–77)	68 (58–81)	58 (52–65)
Missing		297	266	31
2nd Trimester	2859	69 (60–80)	72 (63–84)	63 (57–71)
Missing		911	838	73
3rd Trimester	2613	72 (64–83)	75 (67–89)	67 (61–75)
Missing		1157	1117	40

a Median (Q1–Q3).

b n (%).

The distribution of neonatal outcomes differed significantly between cohorts (Table 2). PTB was more frequent in Araraquara (8.4%) than in Jundiaí (4.9%) (p < 0.001). Similarly, LBW occurred more often in Araraquara (13.0%) compared with Jundiaí (6.3%) (p < 0.001). In contrast, macrosomia was more prevalent in Jundiaí (8.1%) than in Araraquara (4.0%) (p < 0.001). The proportion of infants with a low 5-min Apgar score (<7) was low and comparable between cohorts (1.2% in Araraquara vs. 1.0% in Jundiaí; p = 0.70).

**Table 2:** Distribution of neonatal outcomes in Araraquara and Jundiai Cohort Studies, São Paulo, Brazil.

Neonatal Outcomes^a^	Total N = 3770	Araraquara N = 2805	Jundiaí N = 965	p-value^b^
Preterm birth				<0.001
Term (≥37 weeks)	2493 (92.9%)	1577 (91.6%)	916 (95.1%)	
Preterm (<37 weeks)	191 (7.1%)	144 (8.4%)	47 (4.9%)	
Missing	1086	1084	2	
Adequate Apgar score				0.7
Low Apgar (<7)	32 (1.1%)	23 (1.2%)	9 (1.0%)	
Adequate Apgar (≥7)	2774 (98.9%)	1859 (98.8%)	915 (99.0%)	
Missing	964	923	41	
Low birth weight				<0.001
Normal weight (≥2500 g)	2565 (89.2%)	1662 (87.0%)	903 (93.7%)	
Low birth weight (<2500 g)	309 (10.8%)	248 (13.0%)	61 (6.3%)	
Missing	896	895	1	
Macrosomia				<0.001
Normal weight (<4000 g)	2720 (94.6%)	1834 (96.0%)	886 (91.9%)	
Macrosomia (≥4000 g)	154 (5.4%)	76 (4.0%)	78 (8.1%)	
Missing	896	895	1	

a Values are presented as absolute numbers (n) and column percentages (%).

b Pearson’s Chi-squared test.

**Fig. 1:**
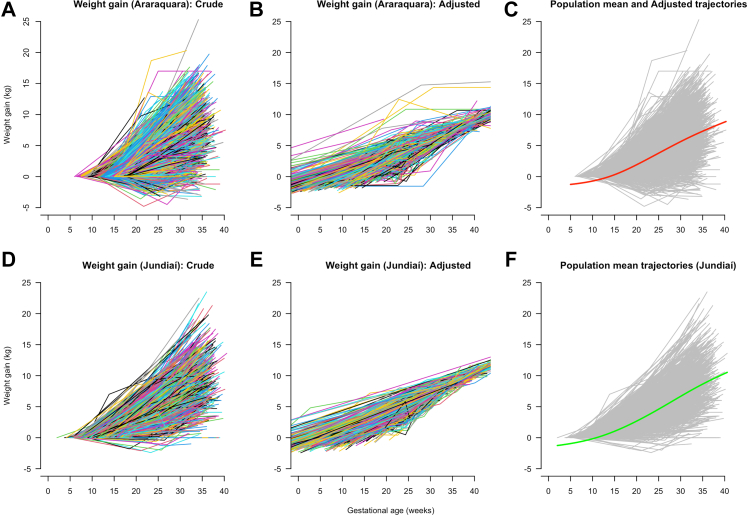
Gestational weight gain trajectories for pregnant women from the Araraquara cohort (Panels A, B, and C) and the Jundiaí cohort (Panels D, E, and F). Panels A and D present the observed (crude) weight gain data; Panels B and E show trajectories adjusted using the SITAR model, accounting for individual variations in size and timing; and Panels C and F display the smoothed fitted individual trajectories together with the population mean trajectory (red line). SITAR, Super-Imposition by Translation and Rotation.

**Fig. 2:**
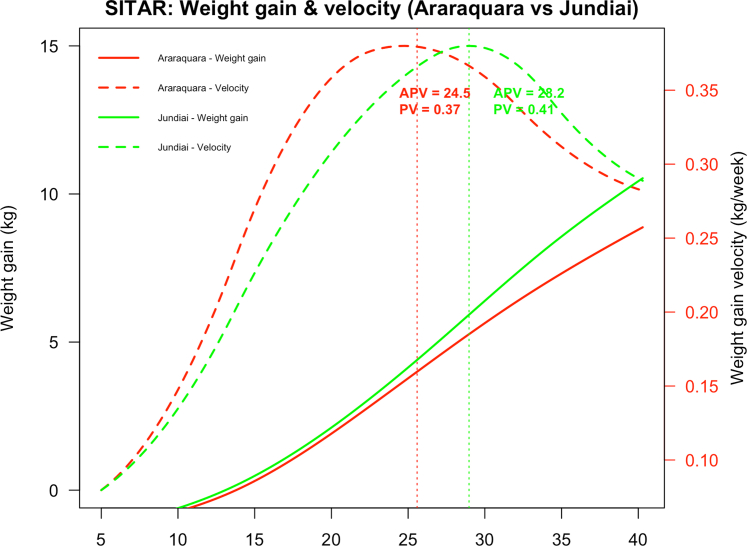
Population-average GWG trajectories (solid lines) and velocities (dashed lines) from the best-fitting SITAR model in Araraquara (red) and Jundiaí (green), Brazil. For Araraquara: APV = 24.5 weeks, PV = 0.37 kg/week, with an estimated total GWG of 8.8 kg at 40 weeks. For Jundiaí: APV = 28.2 weeks, PV = 0.41 kg/week, with an estimated total GWG of 10.3 kg at 40 weeks. These models include random effects for amount and acceleration but do not include a fixed effect.

As shown in Table 3, Supplementary Fig. S3 and Figs. 1 and 2, the Araraquara model estimated the age at peak velocity (APV) at 24.5 weeks and the peak velocity (PV) at 0.37 kg/week, while in Jundiaí, the APV was 28.2 weeks, and the PV was 0.41 kg/week. Both models showed good fit, explaining 84.0% and 85.6% of the variance in GWG, respectively.

**Table 3:** Comparison of SITAR models applied to GWG in two Brazilian Cohorts (Jundiaí and Araraquara) with measurements between 10 and 40 weeks of gestation.

Cohort	Model	Random effects	AIC	BIC	APV	PV	Variance explained (%)
Araraquara	Amount + acceleration	Size, Velocity	23866.2	23933.6	24.48	0.37	84.02
Jundiai	Amount + acceleration	Size, Velocity	10809.7	10868.8	28.19	0.41	85.61

Note: All models were fitted with 4 degrees of freedom (df = 4).

AIC = Akaike Information Criterion, BIC = Bayesian Information Criterion, APV = age at peak velocity, PV = peak velocity.

### Association between SITAR-derived GWG random effects and birth outcomes

According to Table 4 and Fig. 2, GWG parameters derived from SITAR-based predictions showed distinct associations with neonatal outcomes. Compared with women whose GWG was between the 25th and 75th centiles, GWG below the 25th centile was associated with a higher risk of LBW (RR 2.14; 95% CI 1.47–3.11; p < 0.001) and PTB (RR 1.95; 95% CI 1.20–3.17; p = 0.007), and with a lower risk of macrosomia (RR 0.47; 95% CI 0.23–0.97; p = 0.041). GWG size above the 75th centile was not associated with LBW or PTB but was associated with a higher risk of macrosomia (RR 2.58; 95% CI 1.40–4.77; p = 0.002).

**Table 4:** Adjusted risk ratios (RRs) with 95% confidence intervals (CIs) for neonatal outcomes according to and GWG parameters (size and velocity) below 25th centile and above the 75th centile of the INTERGROWTH-21st (IG) standard in reference to women gaining weight between 25th and 75th centile GWG, in the overall sample.

Predictors	LBW			PTB			Macrosomia			Apgar <7		
	RR	95% CI	p	RR	95% CI	p	RR	95% CI	p	RR	95% CI	p
GWG between 25th and 75th centile IG standard	1	ref	ref	1	ref	ref	1	ref	ref	1	ref	ref
GWG below 25th centile IG standard	2.14	1.47–3.11	**<0.001**	1.95	1.20–3.17	**0.007**	0.47	0.23–0.97	**0.041**	1.01	1.00–1.02	0.180
GWG above 75th centile IG standard	0.68	0.31–1.51	0.347	1.15	0.51–2.55	0.740	2.58	1.40–4.77	**0.002**	1.01	1.00–1.02	**0.190**
GWG velocity between 25th and 75th centile IG standard	1	ref	ref	1	ref	ref	1	ref	ref	1	ref	ref
GWG velocity below 25th centile IG standard	2.00	1.20–3.33	**0.008**	1.36	0.75–2.45	0.309	0.63	0.30–1.35	0.238	1.00	0.99–1.02	0.535
GWG velocity above 75th centile IG standard	0.91	0.42–1.97	0.820	1.22	0.55–2.71	0.629	3.31	1.58–6.92	**0.002**	1.01	1.00–1.02	0.099

Risk ratios were estimated using robust Poisson regression, adjusted for maternal age, pre-pregnancy BMI, education, diabetes, smoking, alcohol use, hypertension, newborn sex and parity. Values in bold indicate statistical significance (p < 0.05).

Reference category: GWG between the 25th and 75th percentiles of the INTERGROWTH-21st standard.

LBW–low birth weight, PTB- preterm birth.

For GWG velocity, values below the 25th centile were associated with a higher risk of LBW (RR 2.00; 95% CI 1.20–3.33; p = 0.008), with no clear associations with PTB, macrosomia, or Apgar <7. In contrast, GWG velocity above the 75th centile was strongly associated with macrosomia (RR 3.31; 95% CI 1.58–6.92; p = 0.002), while showing no statistically significant associations with LBW, PTB, or Apgar <7.

When stratified by cohort (Supplementary Table S1), the direction of associations was broadly consistent, although effect sizes differed. In Araraquara, GWG below the 25th centile was associated with a higher risk of LBW (RR 2.03; 95% CI 1.29–3.19; p = 0.002), and low GWG velocity (<P25) was also strongly associated with LBW (RR 3.11; 95% CI 1.46–6.61; p = 0.003). In Jundiaí, GWG below the 25th centile was associated with higher risks of LBW (RR 2.42; 95% CI 1.27–4.58; p = 0.007) and PTB (RR 2.61; 95% CI 1.25–5.47; p = 0.011), and with a lower risk of macrosomia (RR 0.17; 95% CI 0.04–0.79; p = 0.024). GWG above the 75th centile was associated with increased risk of macrosomia (RR 2.82; 95% CI 1.22–6.51; p = 0.015). For GWG velocity, high velocity (>P75) was strongly associated with increased risk of macrosomia (RR 4.10; 95% CI 1.50–11.25; p = 0.006).

## Discussion

This study demonstrated that simplified SITAR models provided robust fits for individual GWG trajectories, explaining more than 84% of the variance in both Brazilian cohorts. SITAR offers an innovative approach by combining a nonlinear spline for the average GWG curve with individual-level random effects that capture deviations in size and velocity.^41^ Unlike traditional mixed models based on random intercepts and slopes, SITAR aligns individual trajectories to the population curve, allowing a more interpretable summary of how women differ in the amount and rate of weight gain over gestation, which may reflect physiological and metabolic variation in GWG dynamics.

Using the intergrowth-21st centiles to interpret predicted GWG, size < P25 was associated with higher risks of LBW and PTB, and a lower risk of macrosomia. GWG size > P75 increased the risk of macrosomia and showed a small increase in 5-min Apgar score <7. For GWG velocity, low velocity (<P25) was associated with increased risk of LBW, while high velocity (>P75) was strongly associated with macrosomia. Stratified analyses showed broadly consistent directions across cohorts, although effect sizes differed.

We observed differences in size and velocity between Araraquara and Jundiaí, which likely reflect underlying sociodemographic, nutritional, and health-related contrasts between the populations, as well as their distinct calendar periods. The earlier timing of peak GWG velocity in Araraquara compared with Jundiaí may reflect temporal changes in maternal health and prenatal care over the last two decades.^51^, ^52^, ^53^ The Jundiaí cohort was conducted about 20 years ago, while the Araraquara cohort is more recent. These differences may also reflect broader transformations in the Brazilian SUS, still consolidating during the Jundiaí period (1997–2000) the national tobacco-control policy was not yet fully implemented, and routine glucose tolerance testing was not consistently available in primary care, likely under-ascertaining gestational diabetes in Jundiaí; hypertensive disorders, long part of prenatal nursing assessment, are expected to have been more reliably identified in both cohorts.^54^^,^^55^ Epidemiological shifts, such as the rising prevalence of overweight and obesity among women of reproductive age, along with increased medicalisation of pregnancy, may have led to earlier weight accumulation during gestation.^53^ Notably, although early-life mortality in Brazil decreased by 25% over the 25 years separating the two cohorts and by 37% in the state of São Paulo, where both were conducted,^54^ national data show that the prevalence of PTB has paradoxically increased over recent decades,^55^ a pattern consistent with the higher PTB prevalence observed in our more recent cohort.

Similar findings were reported by Riddell et al. (2017), who also encountered convergence issues when applying the full SITAR model with all random effects specified. As in our study, these authors opted for simplified versions of the model, which showed better performance and successfully described GWG trajectories. In their analysis of a cohort of 3470 women in the United States, the reduced models explained between 95% and 97% of the variance in GWG trajectories, effectively capturing the absolute amount and velocity of weight gain during pregnancy.^48^ This two-parameter SITAR specification is also consistent with applications beyond pregnancy; Ohuma and his colleagues (2021) used the same two-parameter structure to model child linear growth across 64 countries and 145 surveys, demonstrating that this reduced specification can capture substantive between-individual variability in growth size and rate even when timing is omitted.^45^ The fit obtained in our cohorts is consistent with these prior applications and supports the adequacy of the reduced specification for trajectory modelling in our data.

The SITAR model may also help in deducing GWG and understanding the rate of weight gain which is important. As with child growth analysis, where heterogeneity in growth velocity is observed, GWG also varies substantially among pregnant women. These variations can be represented by the SITAR parameters: α (size), which defines the woman’s average weight throughout pregnancy, adjusting for individual differences in baseline weight; which reflects individual shifts in GWG trajectories, indicating when a woman gains weight more rapidly, possibly linked to metabolism, eating habits, and pregnancy complications; and c (velocity), which indicates the rate of weight gain during pregnancy. As in pubertal growth velocity, variation in this parameter may reflect differences in metabolism, pre-pregnancy nutritional status, and risk of complications such as fetal growth restriction or macrosomia. SITAR analyses indicate that SITAR parameterisation may aid in identifying atypical weight gain trajectories and potential maternal–fetal risks, characterising individual patterns to predict adverse outcomes such as PTB or inadequate fetal growth, and distinguishing women who gain weight more rapidly at different gestational stages, thereby supporting personalised interventions.^10^^,^^35^^,^^41^^,^^48^^,^^56^

This study has several strengths. To our knowledge, it is the first to apply the SITAR model to GWG trajectories in two population-based Brazilian cohorts with distinct temporal and epidemiological contexts. The use of harmonised data allowed for robust comparisons, and the SITAR model explained over 84% of the variance in GWG using only *size* and *velocity* parameters. Moreover, the study went beyond descriptive modelling and explored associations between SITAR-derived parameters and neonatal outcomes, revealing consistent patterns of association with macrosomia and low birth weight. These findings support the clinical relevance of GWG dynamics and illustrate the potential of SITAR modelling to inform risk stratification and maternal health interventions.

However, some limitations should be acknowledged. First, GWG is an indirect marker of maternal nutrition and body composition and is subject to measurement error from self-reported pre-pregnancy weight. Second, the SITAR model is statistically demanding and may require variable transformations or structural simplifications to converge,^41^ and the interpretation of certain parameters in the GWG context remains unclear.^48^ For example, the size parameter is of limited use in GWG, as all women should start pregnancy with zero weight gain, but it may partially capture measurement error from self-reported pre-pregnancy weight. Third, the two cohorts differ in important methodological aspects that may affect comparability they were conducted two decades apart, under different prenatal care protocols, with heterogeneous gestational age dating and varying frequency and standardisation of weight measurements. Fourth, SGA and LGA were excluded because gestational age was estimated differently across cohorts. Finally, the absence of reference standards to interpret SITAR parameters in relation to neonatal outcomes leaves their interpretation exploratory; residual confounding by dietary intake and metabolic status cannot be excluded. Future research should integrate SITAR modelling with established GWG references to improve interpretability and clinical relevance; evaluate alternative approaches, such as group-based trajectory modelling, in cohorts with denser longitudinal data; extend the analysis to additional neonatal outcomes in contemporary cohorts; and develop BMI-specific GWG references.

### Conclusion

This study demonstrated that the SITAR model is a useful approach for characterising GWG trajectories in two distinct Brazilian cohorts, explaining more than 84% of the variability in GWG. When SITAR-predicted GWG size and velocity were classified using IG centiles, clear risk patterns emerged, GWG size < P25 was associated with higher risks of LBW and PTB and a lower risk of macrosomia, whereas GWG size > P75 and especially GWG velocity > P75 were associated with increased risk of macrosomia. The 25th and 75th centiles of IG-21st for GWG among women with normal weight can provide a risk stratification that is consistent with identifying those mostly at risk of poor neonatal outcomes and inform more targeted monitoring and interventions in prenatal care.

## Contributors

Conceptualization, formal analysis, methodology: A.V., P.H.C.R., and E.O.O.

Data curation: A.V. and E.O.O.

Funding acquisition, supervision: P.H.C.R. and L.A.L.

Investigation: A.V.,T.R.S.,I.O.A., A.P.L, R.E.P., and E.O.O.

Project administration: P.H.C.R.

Visualisation, writing and original draft: A.V., P.H.C.R., and E.O.O.

Writing, review & editing: All authors contributed to and approved the final version of the article. A.V., P.H.C.R., and E.O.O accessed and verified the underlying data. All authors had full access to the study data and had final responsibility for the decision to submit it for publication.

## Data sharing statement

The R code used for data preparation, SITAR modelling, INTERGROWTH-21st classification, and Poisson regression analyses is publicly available at https://github.com/Audency/gwg-sitar-cohorts. The data supporting the conclusions of this article will be made available by Professor Patrícia Helen Rondó, without undue reservation.

## Declaration of interests

The authors declare that they have no conflicts of interest.
