# Supplementary material for: Modelling gestational weight gain trajectories and risk of adverse birth outcomes using super imposition by translation and rotation: findings from two Brazilian cohort studies
Source: Lancet Reg Health Am. 2026 Jul 9;62:101561. doi: 10.1016/j.lana.2026.101561 (PMC13377129; doi:10.1016/j.lana.2026.101561)
Supplement: Supplementary Figs. S1-S3 and Tables S1 and S2 [file mmc1.pdf]

Revised manuscript (with marked changes) Click here to view linked References

Figure S1. Cumulative gestational weight gain – Cohort comparison between Jundiaí and Araraquara (Gestational age between 16 and 40 weeks).

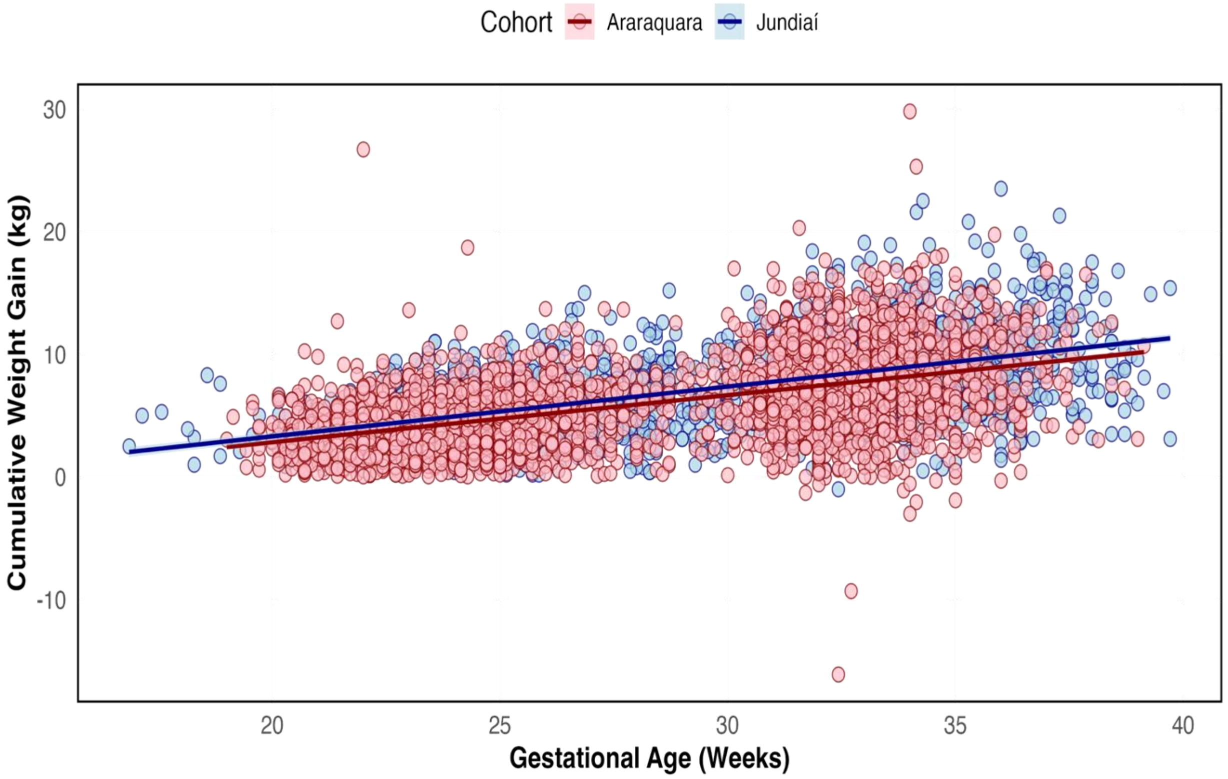

Figure S2. Cumulative gestational weight gain – Cohort comparison between Jundiaí and Araraquara (Gestational age between 16 and 40 weeks) – Smoothed percentiles.

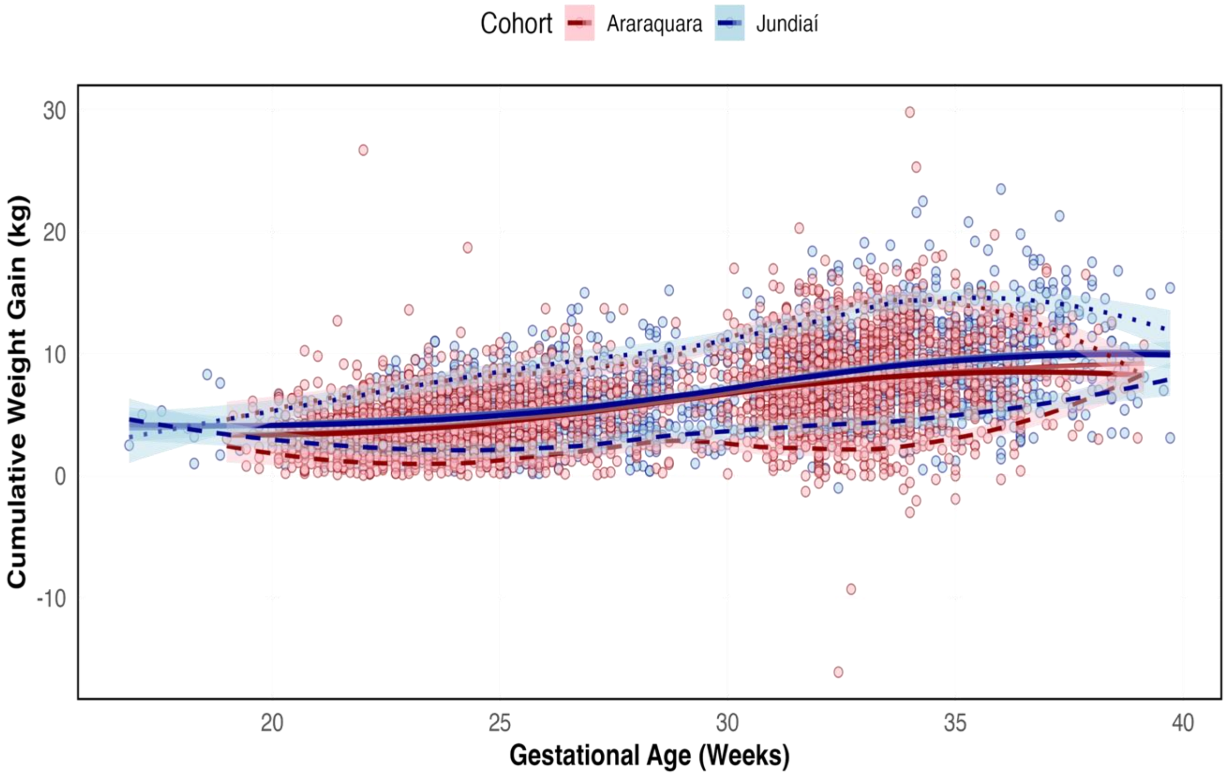

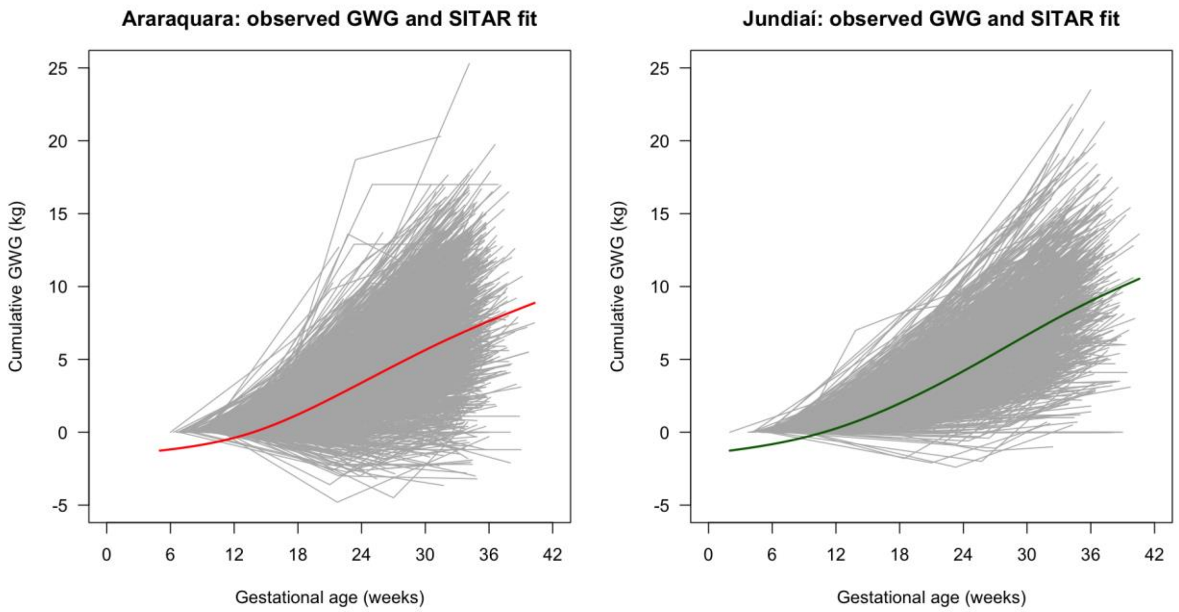

Fig. S3. GWG trajectories and SITAR population mean fit in the Araraquara and Jundiaí cohorts. Grey lines represent individual observed GWG trajectories, and the coloured line represents the population mean trajectory estimated by the SITAR model (red for Araraquara and green for Jundiaí). SITAR, Super-Imposition by Translation and Rotation.

Table S1. Adjusted risk ratios (RRs) with 95% confidence intervals (CIs) for neonatal outcomes according to and GWG parameters (size and velocity) below 25th centile and above the 75th centile of the INTERGROWTH-2^{1st} (IG) standard in reference to women gaining weight between 25th and 75th centile GWG, stratified by cohort.

	Araraquara								Jundiaí							
**Predictors**	**LBW RR (95% CI)**	**p**	**PTB RR (95% CI)**	**p**	**Macrosomia RR (95% CI)**	**p**	**Apgar <7 RR (95% CI)**	**p**	**LBW RR (95% CI)**	**p**	**PTB RR (95% CI)**	**p**	**Macrosomia RR (95% CI)**	**p**	**Apgar <7 RR (95% CI)**	**p**
GWG between 25th and 75th centile IG standard	1	—	ref	—	ref	—	ref	—	ref	—	ref	—	ref	—	ref	—
GWG below 25th centile IG standard	**2.03 (1.29–3.19)**	**0.002**	1.45 (0.78–2.69)	0.241	0.70 (0.28–1.73)	0.440	1.01 (0.99–1.02)	0.284	**2.42 (1.27–4.58)**	**0.007**	**2.61 (1.25–5.47)**	**0.011**	**0.17 (0.04–0.79)**	**0.024**	1.01 (0.99–1.02)	0.434
GWG above 75th centile IG standard	0.88 (0.37–2.10)	0.775	0.86 (0.29–2.55)	0.782	1.54 (0.55–4.35)	0.412	1.01 (1.00–1.02)	0.113	0.28 (0.04–2.19)	0.227	1.54 (0.48–4.93)	0.471	**2.82 (1.22–6.51)**	**0.015**	1.01 (1.00–1.02)	0.108
GWG velocity between 25th and 75th centile IG standard	1	—	ref	—	ref	—	ref	—	ref	—	ref	—	ref	—	ref	—
GWG velocity below 25th centile IG standard	**3.11 (1.46–6.61)**	**0.003**	1.54 (0.65–3.63)	0.326	0.68 (0.23–1.99)	0.481	1.00 (0.98–1.02)	0.962	1.19 (0.59–2.41)	0.629	1.20 (0.52–2.78)	0.662	0.56 (0.19–1.65)	0.295	1.01 (0.99–1.03)	0.418
GWG velocity above 75th centile IG standard	1.68 (0.64–4.40)	0.295	1.37 (0.46–4.04)	0.573	2.20 (0.74–6.60)	0.158	1.01 (0.99–1.02)	0.418	0.22 (0.03–1.74)	0.152	1.14 (0.33–3.98)	0.837	**4.10 (1.50–11.25)**	**0.006**	1.02 (0.99–1.04)	0.156

Risk ratios were estimated using robust Poisson regression, adjusted for maternal age, pre-pregnancy BMI, education, diabetes, smoking, alcohol use, hypertension, newborn sex and parity

Reference category: GWG between the 25th and 75th percentiles of the INTERGROWTH-21st standard.

LBW - low birth weight, PTB- preterm birth

Table S2. Abbreviations used in the manuscript and supplementary material

Abbreviation	Full Term
**AIC**	Akaike Information Criterion
**APV**	Age at Peak Velocity
**BIC**	Bayesian Information Criterion
**BMI**	Body Mass Index
**CI**	Confidence Interval
**DAG**	Directed Acyclic Graph
**df**	Degrees of Freedom
**GA**	Gestational Age
**GWG**	Gestational Weight Gain
**HC0**	Heteroskedasticity-Consistent Variance Estimator (sandwich)
**IG-21st**	INTERGROWTH-21st gestational weight gain standard
**IOM**	Institute of Medicine
**IQR**	Interquartile Range
**LBW**	Low Birthweight (< 2,500 g)
**P25**	25th Centile
**P75**	75th Centile
**PTB**	Preterm Birth (gestational age < 37 completed weeks)
**PV**	Peak Velocity
**RR**	Risk Ratio
**SD**	Standard Deviation
**SE**	Standard Error
**SITAR**	Super-Imposition by Translation and Rotation (nonlinear mixed-effects growth curve model)
**SUS**	Sistema Único de Saúde (Brazilian Unified Health System)

All analyses were performed in R v4·1·0 using the sitar (v1·0·3), sandwich, lmtest,
